# Molecular understanding and clinical aspects of tumor-associated macrophages in the immunotherapy of renal cell carcinoma

**DOI:** 10.1186/s13046-024-03164-y

**Published:** 2024-08-22

**Authors:** Han Liu, Zongwei Lv, Gong Zhang, Zhenhong Yan, Song Bai, Dan Dong, Kefeng Wang

**Affiliations:** 1 Department of Urology, Shengjing Hospital of China Medical University, #36 Sanhao Street, Shenyang, Liaoning 110004 China; 2 College of Basic Medical Science, China Medical University, #77 Puhe Road, Shenyang, Liaoning 110122 China

**Keywords:** Immunotherapy, Renal cell carcinoma, Tumor-associated macrophages, Tumor microenvironment

## Abstract

Renal cell carcinoma (RCC) is one of the most common tumors that afflicts the urinary system, accounting for 90–95% of kidney cancer cases. Although its incidence has increased over the past decades, its pathogenesis is still unclear. Tumor-associated macrophages (TAMs) are the most prominent immune cells in the tumor microenvironment (TME), comprising more than 50% of the tumor volume. By interacting with cancer cells, TAMs can be polarized into two distinct phenotypes, M1-type and M2-type TAMs. In the TME, M2-type TAMs, which are known to promote tumorigenesis, are more abundant than M1-type TAMs, which are known to suppress tumor growth. This ratio of M1 to M2 TAMs can create an immunosuppressive environment that contributes to tumor cell progression and survival. This review focused on the role of TAMs in RCC, including their polarization, impacts on tumor proliferation, angiogenesis, invasion, migration, drug resistance, and immunosuppression. In addition, we discussed the potential of targeting TAMs for clinical therapy in RCC. A deeper understanding of the molecular biology of TAMs is essential for exploring innovative therapeutic strategies for the treatment of RCC.

## Introduction

Renal cell carcinoma (RCC) is one of the most common malignant tumors of the urinary system, accounting for approximately 90–95% of all kidney cancer cases [1]. Approximately 400,000 RCC patients are discovered worldwide each year, and the annual growth rate is around 2% [2]. Surgical operation is a common method that achieves good prognosis for patients in the early stage of RCC treatment [3]. Unfortunately, up to one-third of RCC patients are at risk of tumor invasion, even after receiving timely diagnosis and treatments [4, 5]. Therefore, a deeper investigation of the mechanisms of RCC development could lead to the discovery of more effective therapies. Non-tumoral components of tumor tissue, such as immune cells, also play an essential role in tumor development and metastasis [1]. Therefore, focusing anti-tumor efforts on the immune components of the tumor microenvironment (TME) may be a viable strategy for future tumor therapy approaches.

Among the multiple immune constituents of the TME, macrophages are the most populous infiltrating immune cells. Tumor microenvironmental cytokines are small soluble proteins that encode instructions and mediate communication among immune and non-immune cells to modulate immune processes. A network of cytokines is an essential component of the role of macrophages as regulators of the innate immune system. Therefore, the interaction of various cytokines with macrophages in the TME has a critical impact on the development and prognosis of RCC [2]. In contrast to the healthy activities of macrophages, macrophage–tumor cell interactions induce changes on macrophage polarization, leading to tumor metastasis and immunosuppressive properties [3, 4]. Macrophages that interact with tumor cells are knows as tumor-associated macrophages (TAMs) and play an important role as the most abundant immune population in the TME. Tissue-specific macrophages that reside in or are recruited into the tumor tissue. Tissue-resident macrophages form during embryotic development, whereas tissue-recruited macrophages form from monocytes, a type of white blood cell that originates in bone marrow and migrates via the bloodstream to peripheral tissues, where they differentiate into macrophages. Due to their complex effects on the TME, TAMs are both targets for immunotherapy and innate sources of therapeutic potency [5, 6].

This review aims to investigate the molecular mechanisms of TAMs in the development of RCC and the potential for their application in RCC immunotherapy, so as to provide ideas for targeted therapy and improve the survival rate of RCC patients.

## TAMs and RCC

### Macrophages and TAMs

Macrophages play a critical role in the body’s innate immune system. In response to infection, macrophages conduct immune defenses against a variety of cellular substrates, mainly through phagocytosis and their antigen presentation capacity [7]. In addition, macrophages have significant functions in tissue repair and homeostasis maintenance [8, 9], with considerable functional plasticity and flexibility, enabling them to adapt to different signaling factors and tissue environments [10]. Macrophages have two main origins: tissue-specific resident macrophages from embryonic precursors of the yolk sac, and tissue-recruited macrophages that differentiate from circulating blood monocytes produced in bone marrow [11]. Peripheral circulating blood monocytes are the most abundant source of macrophage recruitment, with a minor contribution coming from tissue-specific resident macrophages.

M0 macrophages are resting-state macrophages that serve as precursors of polarized macrophages, M1 and M2 macrophages generated from M0. M0 has no specific function other than to polarize into M1 or M2 macrophages. The M1 and M2 (M1/2) subtypes are macrophages that perform two dual-polarized activities: M1 activity inhibits cell proliferation and causes tissue damage, whereas M2 activity promotes cell proliferation and tissue repair. M0 macrophages that polarize into TAMs have two major sources, those present in tumor tissue and those recruited to tumor tissue by chemokines and cytokines. M0 TAMs are primarily activated to anti-tumor and pro-tumor phenotypes under the action of various cytokines or metabolites derived from tumor tissues. The anti-cancer subtype M1 TAMs, known as “killer macrophage” or “classically activated macrophage,” have a phagocytic capacity and act as a pro-inflammatory and anti-infective agent. The pro-oncogenic subtype M2 TAMs, called “repair macrophages,” are alternatively activated to alleviate the inflammatory status and perform anti-inflammatory functions [12]. Although the M1/2 classification is oversimplified, it explains the opposing cancer-promoting and cancer-fighting properties, contributing to an improved understanding of the polarized roles of M1 and M2 TAMs in tumorigenesis, angiogenesis, progression, and metastasis. In addition, more detailed subtype classifications for different characteristics are being standardized [13].

**Fig. 1:**
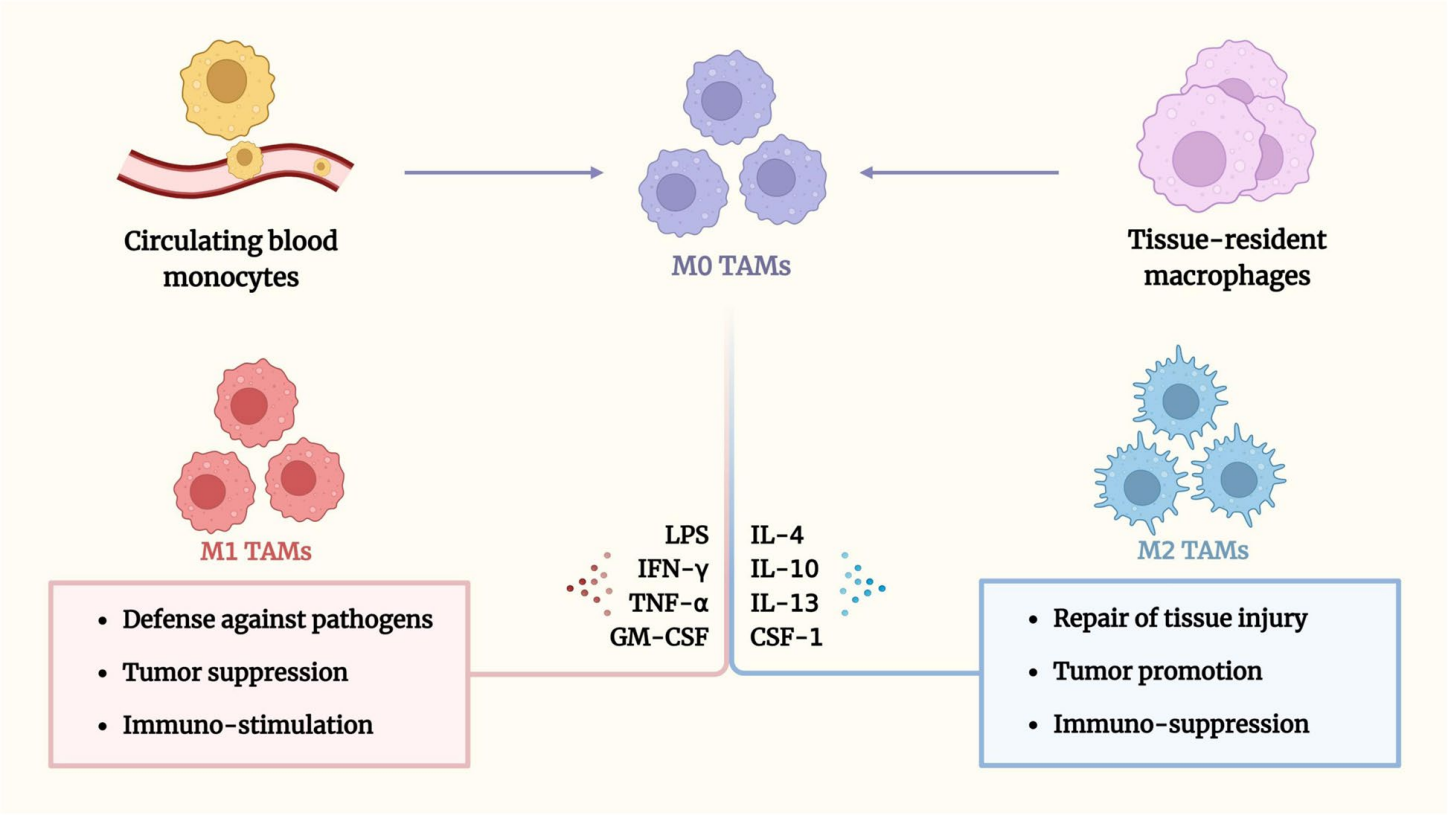
Sources and polarization of TAMs. There are two main sources of recruitment for M0 TAMs: monocytes from circulating blood and tissue-resident macrophages. Once recruited into the TME, M0 TAMs differentiate and mature into M1 TAMs (typically activated macrophages) in the presence of LPS, IFN-γ, TNF-α, and GM-CSF. In contrast, M0 TAMs are polarized into M2 TAMs (alternatively activated macrophages) under the action of IL-4, IL-10, IL-13, and CSF-1. M1 TAMs play important roles in immunostimulatory and anti-tumor processes. M2 TAMs, on the other hand, generally exhibit tumor-promoting phenotype and immunosuppressive effects that contribute to anti-inflammatory and tissue repair processes

TAMs develop the M1/2 phenotype under the influence of diverse factors and have great functional plasticity (Fig. 1). M0 TAMs are activated into typical M1 TAMs by appropriate concentrations of lipopolysaccharide (LPS), interferon-γ (IFN-γ), tumor necrosis factor-α (TNF-α), and granulocyte-macrophage colony stimulating factor (GM-CSF) [14]. As immune stimulators, M1 TAMs participate in pathogen defenses, phagocytic processes, and intrinsic and adaptive immune responses [15]. They secrete anti-angiogenic or immunostimulatory cytokines and chemokines to stimulate inflammatory responses, including TNF-α, interleukin (IL)-1β, IL-2, IL-6, IL-12, and IL-23, and may also act as tumor-suppressors [16, 17]. They express CD80 and CD86 molecules (B7 family members), triggering helper T cell type 1 immune responses to clear sick cell types [18]. However, the persistent activity of M1 TAMs in the inflammation foci may injure the tissue and impede wound healing.

Conversely, M0 TAMs are activated into M2 TAMs by IL-4, IL-10, IL-13, CSF-1, corticosteroids, and other factors [17, 19], including certain immune cells, specific genetic oncogenes, and lactic acid-rich hypoxic conditions that promote M2 TAM polarization through different mechanisms [20, 21]. M2 TAMs, which are mainly involved in tissue repair and phagocytosis, typically exhibit tumor-promoting phenotype and immunosuppressive effects that promote tumor development and contribute to tissue repair processes and express high levels of anti-inflammatory and tumor angiogenesis factors: IL-10, programmed death-ligand 1 (PD-L1), transforming growth factor-β (TGF-β), vascular endothelial growth factor (VEGF), some growth factors, different scavenger receptors (CD163 and CD204), and arginase-1 [22, 23]. These agents facilitate pro-tumorigenic processes and accelerate tumor development and metastasis. Simultaneously, the amplified Th2 response by M2 TAMs balances indiscriminate tissue damage caused by the Th1 response [24], while the growth factors produced by the Th2 response further mobilize the activation of M2 TAMs [25].

To sum up, TAMs are clearly a double-edged sword, processing both tumor-promoting and cancer-fighting properties in the TME. Unfortunately, M2 TAMs dominate the TME, which encourages tumor progression and aggravation, but serve as a potential therapeutic target or biomarker for tumor-targeted immunotherapy.

In addition to the regulatory mechanisms of cytokines and chemokines, several metabolites and metabolic changes in the TME also interact with the polarization of TAMs [26]. M1 TAM polarization is associated with an increased rate of LPS-induced aerobic glycolysis. Glycolysis promotes the increase of carbon flux into the oxidized pentose phosphate pathway, thus accelerating the production of reactive oxygen species (ROS) in M1 macrophages [27], which is further facilitated by pro-inflammatory metabolites. M1 TAMs continuously accumulate succinate during the interrupted tricarboxylic acid (TCA) cycle [28] and stabilize hypoxia-inducible factor 1α (HIF-1α) through inhibition of prolyl hydroxylase activity, which is crucial for ROS production [29]. Therefore, M1 TAMs can generate a large number of ROS through metabolic pathways, induce a highly positive oxidative environment, and maintain the pro-inflammatory state of macrophages [22].

On the contrary, fatty acid oxidation (FAO) becomes an important energetic source for pro-tumor TAM polarization due to the low rate of aerobic glycolysis in M2 TAMs. Specifically, IL-4 induces M0 TAM polarization towards M2 TAMs, promotes fatty acid uptake and oxidation, and enhances mitochondrial biogenesis [30]. Similar to M1 TAMs, M2 TAM polarization also relies on a key metabolite of the TCA cycle. α-Ketoglutarate promotes FAO and the epigenetic regulation of Jumonji structural domain-containing protein 3 (JMJD3) for M2 gene expression, which is important for M2 TAM activation [29]. Moreover, α-ketoglutarate can destabilize HIF-1α, thereby restricting M1 TAMs activation. It is clear that TAMs play a predominant role in the RCC immune microenvironment (IME) and a key role in RCC development. A thorough comprehension of the interactions among TAMs and RCC cells is essential to advance immunotherapies and clinical applications for urinary system tumors.

### Interactions between TAMs and RCC

TAMs hold a significant position in the IME of RCC, and their influence is crucial in the onset and progression of RCC. A clear investigation of the interactions between TAMs and RCC cells could lead to the development of novel targeted and immunotherapeutic strategies. This section details the interactions between TAMs and RCC cells, focusing on the polarization of TAMs and their various impacts on the progression of RCC, including proliferation, angiogenesis, invasion, immune suppression, and therapy resistance.

#### Polarization of TAMs in RCC

As previously discussed, the two major polarized subtypes of M0 TAMs in RCC, M1 and M2 TAMs, play diametrically opposed roles in tumor progression. The infiltration of M1 TAMs is negatively associated with tumor metastasis and the TNM Classification of Malignant Tumors (TNM) grade, and a higher infiltration of M1 TAMs has been linked to a higher survival rate in RCC patients [2]. Nonetheless, M2 TAMs dominate the major subtypes of TAMs and are related to poorer prognosis in RCC [31]. M2 TAMs could secret C-X-C motif chemokine ligand 13 (CXCL13), thus facilitating RCC invasion, migration, and epithelial-mesenchymal transition (EMT) [32]. In addition, infiltration of M2 TAMs has been associated with RCC recurrence [33]. These findings may herald M2 TAMs as a potential target for immunotherapy in RCC.

The interplay between TAMs and their surrounding tumor IME is intricate, involving multiple molecular pathways and factors. Exosomes are critical mediators in the interplay between RCC cells and the immune response. Importantly, exosomes are thought to exert an important role in the oncogenic polarization of macrophages mediated by RCC cells [34]. Long non-coding RNAs (lncRNAs) contained in exosomes from RCC cells can promote M2 TAM polarization through the signal transducer and activator of transcription 3 (STAT3) signaling pathways. LncARSR directly interacts with miR-34/miR-449, increases the STAT3 expression, and mediates TAM polarization in the RCC TME [35]. Similarly, exosomes-loaded lncRNA AP000439.2 promotes STAT3 phosphorylation in TAMs, thus activating the NF-κB signaling pathway and promoting M2 TAM polarization [36]. Circular RNAs (circRNAs) are also involved in M2 TAM polarization. RCC-derived exosomal circSAFB2 induces M2 TAM polarization of macrophages by regulating the JAK1/STAT3 axis through miR-620 [37]. Considered together, these studies indicate that RCC-derived exosomes and the STAT3 pathway play essential roles in activating M2 macrophage polarization, making them potential targets for RCC immunotherapy.

Besides exosomes, a variety of oncogenes and proteins are involved in tumor-coordinated M2 TAM polarization. Transcription factor forkhead box (FOX) k1, which is member of the FOX protein family, is upregulated in RCC and associated with M2 TAM infiltration in RCC [38]. Tumor necrosis factor receptor-associated factor-2 (TRAF2), a member of the TRAF superfamily of proteins that acts as an oncogene, affects tumor development by multiple mechanisms. Knockdown of TRAF2 inhibits M2 TAM polarization through an autophagy-dependent pathway [39]. Another oncogene in RCC is RNA-binding motif protein 15 (RBM15), a component of the methyltransferase complex, which mainly plays a carcinogenic role in various tumors. RBM15 regulates CXCL11 mRNA stability in an m6A-dependent manner, and in vivo and in vitro studies have found that RBM15 promotes infiltration of TAMs and polarization of M2 TAMs by facilitating CXCL11 production by RCC cells [40]. Several other proteins have potential regulatory roles in M2 TAM polarization in RCC cells, including leukotriene B4 receptor 2 (BLTR2), contactin-associated protein 1 (CNTNAP1), and aquaporin 9 (AQP9) [41]. Although these proteins may have a strong correlation with M2 TAM polarization, more intensive research is needed to elucidate their specific functions.

Apart from their induction from cancer cells, M2 TAMs themselves also promote the polarization of TAMs towards the M2 subtype. Apolipoprotein C1 (APOC1) is a protein component of lipoprotein, the expression of which was shown to be remarkably upregulated in M2 TAMs [42]. APOC1 has been associated with the metastasis and prognosis of diverse cancers, and promotes M2 TAM polarization through interaction with CD163 and CD206 [43].

In addition to promoting M2 TAM polarization, RCC cells also inhibit M2 TAMs. miRNA let-7d has been found to be frequently downregulated in various cancers, including RCC, suggesting its pivotal role in tumorigenesis. Moreover, high-density infiltration of TAMs in RCC has been inversely correlated with let-7d expression [44]. Furthermore, overexpression of let-7d in RCC cells was found to inhibit intertumoral M2 TAM polarization and consequent tumor angiogenesis by targeting IL-10 and IL-13 [45]. The polarization is also influenced by the inflammasome component of macrophages, which induces the conversion of M2 TAMs into M1 TAMs. Absent in melanoma 2 (AIM2) deletion is a critical mediator of the immune response, which promotes macrophage function and is involved in RCC cell invasion, migration, and tumor development. Studies have shown that the expression of AIM2 in TAMs correlates negatively with the infiltration of M2 TAMs, and the upregulation of AIM2 reverses the metastasis of M2 TAMs to M1 TAMs induced by RCC cells [46]. Therefore, AIM2-mediated polarization of TAMs may be a new direction for RCC immunotherapy.

#### Angiogenesis and proliferation of RCC affected by TAMs

**Fig. 2:**
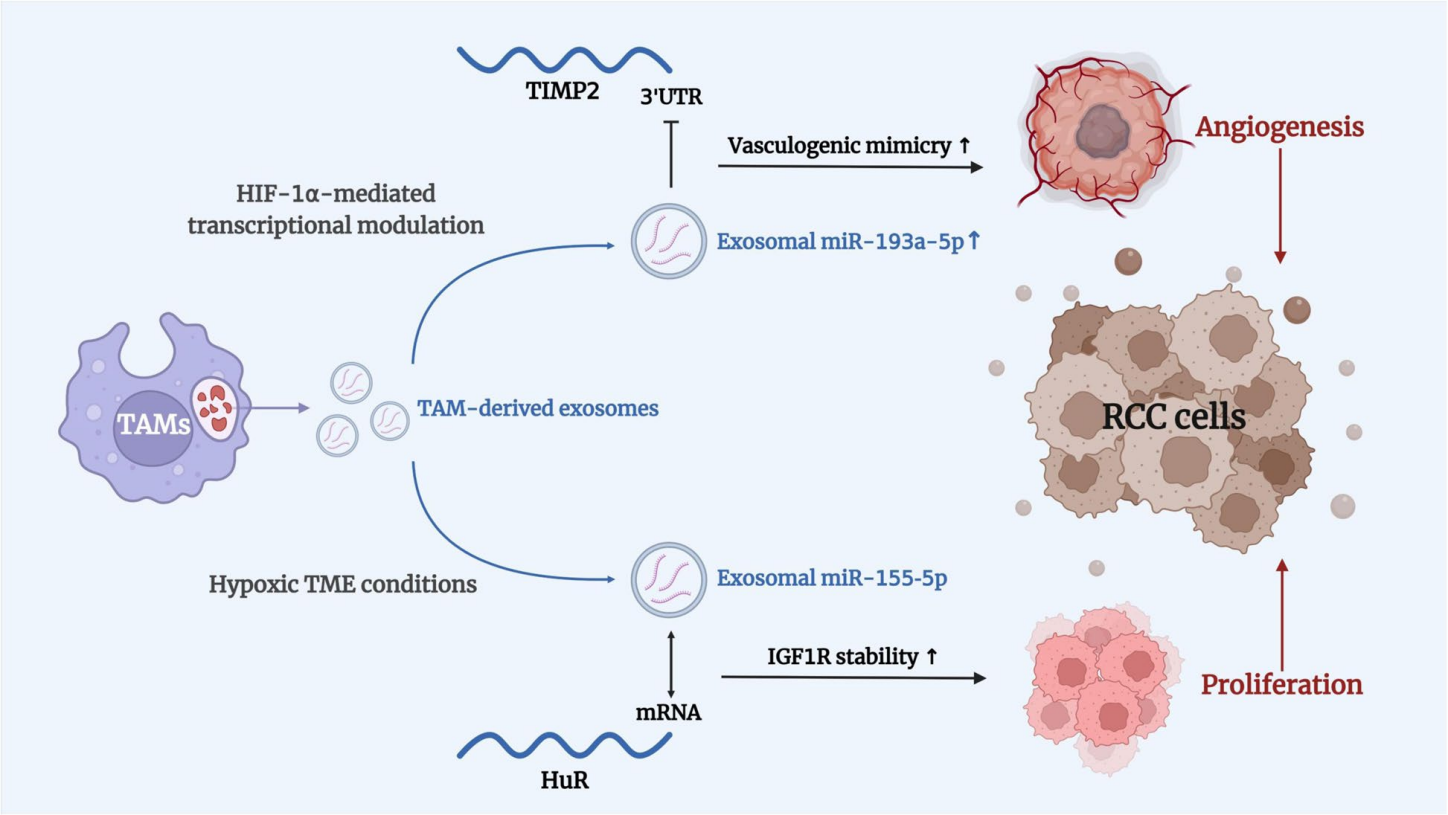
TAMs promote RCC angiogenesis and proliferation. TAM-derived exosomes promoted RCC angiogenesis and proliferation by assembling different miRNAs. Overexpression of miR-193a-5p in exosomes was induced by HIF-1α-mediated transcriptional regulation. Upregulation of miR-193a-5p would inhibit the 3′UTR of TIMP2 mRNA, thereby promoting vasculogenic mimicry and angiogenesis in RCC. Under hypoxic TME conditions, another exosome-loaded miR-155-5p promoted the proliferation of RCC cells. Exosomal miR-155-5p directly interacted with HuR mRNA and promoted RCC cell proliferation by increasing the stability of IGF1R mRNA

Ongoing angiogenesis is an important characteristic of cancer progression. Neovascularization promotes cancer cell proliferation, metastasis, and invasion during the processs of tumorigenesis and progression. Abnormal angiogenesis in solid tumors leads to an imbalance between the tumor growth rate and angiogenesis, resulting in a long-term hypoxic state for the TME. Hypoxia is also closely associated with tumor proliferation and invasion. In the TME, interactions between RCC cells and TAMs not only promote macrophage recruitment and polarization but also facilitate tumor cell proliferation and progression (Fig. 2).

In the RCC TME, some TAM-derived exosomes are closely related to angiogenesis and hypoxia. Exosomes act as important connectors in the interactions between RCC growth and immune cell infiltration, which promote RCC cell growth, development, and metastasis. circRNAs or miRNAs in RCC-loaded exosomes induce M2 TAM chemotaxis and polarization, and in turn, TAM-secreted exosomes promote RCC cell proliferation and angiogenesis. The study revealed that miR-193a-5p in TAM-derived exosomes could facilitate RCC proliferation and angiogenesis by promoting vasculogenic mimicry (VM) and cell invasion [47]. TAM-derived exosomes encapsulate miR-193a-5p and upregulate its expression through HIF-1α-mediated transcriptional modulation. Overexpression of miR-193a-5p could downregulate VM-related genes and promote angiogenesis. Exosome-loaded miR-193a-5p then targets the 3’ untranslated region (UTR) of tissue inhibitor of metalloproteinases 2 (TIMP2) mRNA, thereby suppressing the process of translation and thus promoting the VM and invasion activities of RCC cells. In addition, inhibition of TAM-derived exosomal miR-193a-5p successfully restrained tumor proliferation and progression [47]. These results may provide a new research direction for the discovery of novel immunotherapies for RCC.

Under hypoxic TME conditions, another type of exosome, derived from hypoxic TAMs, is related with the malignant phenotype of RCC cells [48]. Hypoxic exosomes deliver miR-155-5p to recipient cancer cells and regulate its related biofunctions. Exosomal miR-155-5p directly interacts with human antigen R (HuR) mRNA, thereby affecting insulin-like growth factor 1 (IGF-1) expression. The binding of transferred miR-155-5p to HuR improves IGF1R mRNA stability, thereby facilitating the proliferation of RCC cells. Moreover, upon stimulation with hypoxic TAM-exosomes, recipient RCC cells display HuR-dependent upregulation of IGF-IR signaling, leading to the mobilization of the PI3K/AKT downstream pathway [48].

These findings indicate that TAMs in the RCC TME secrete diverse exosomes and are involved in the proliferation and angiogenesis of renal cancer tissues through various homologous pathways.

#### Migration, invasion, and metastasis of RCC affected by TAMs

**Fig. 3:**
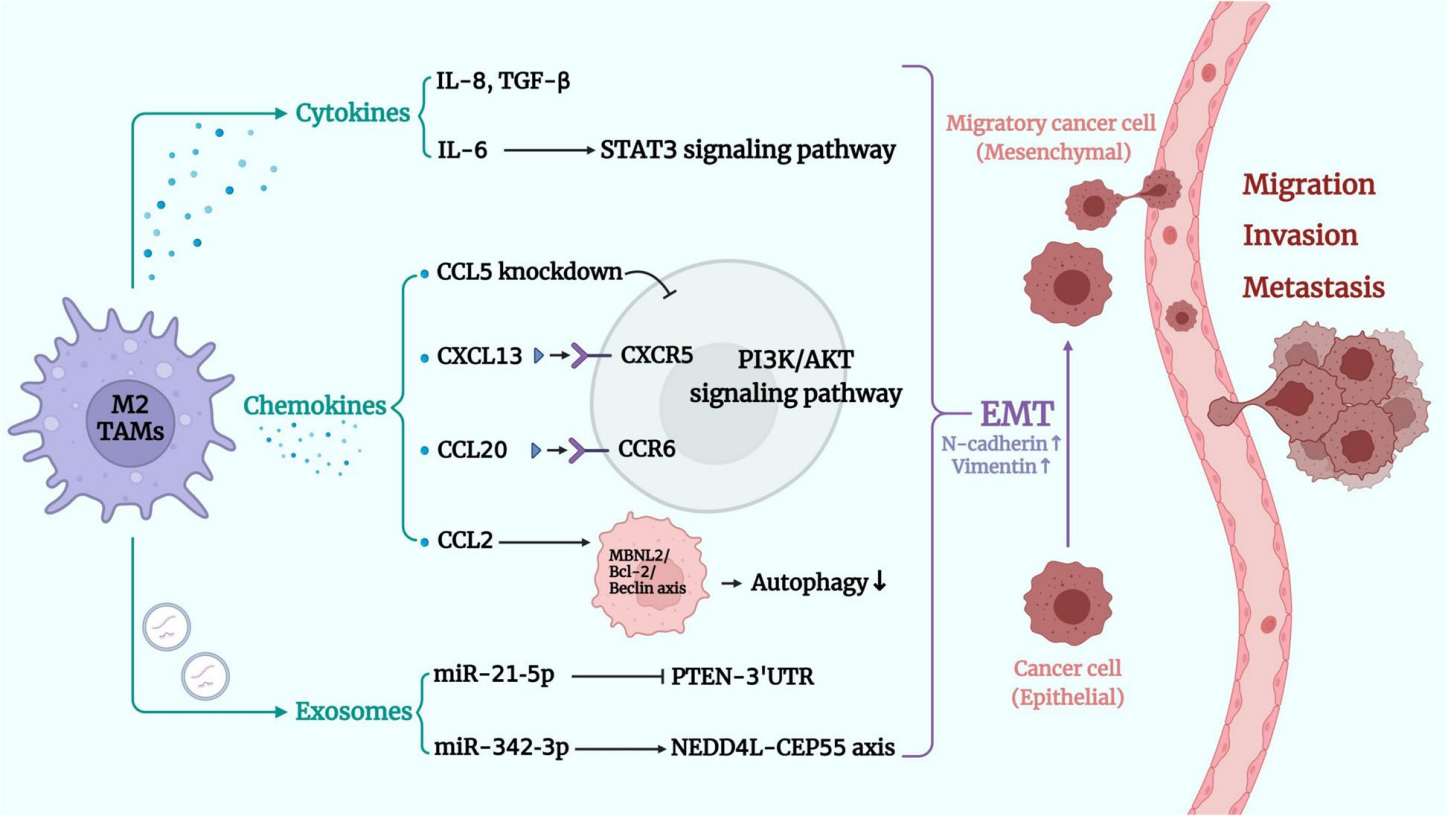
TAMs promote RCC migration, invasion, and metastasis. In the TME of RCC, cytokines, chemokines, and exosomes produced by M2 TAMs play a critical role in regulating migration, invasion, and metastasis of RCC cells. First, M2 TAMs secrete a variety of cytokines, including IL-6, IL-8, and TGF-β, inducing tumor cells to develop EMT and promoting tumor metastasis. Among them, IL-6 can promote RCC cell migration, invasion and EMT by activating STAT3 signaling pathway. Secondly, various chemokine ligands of M2 TAMs can affect EMT in RCC TME. CCL5 knockdown prevents EMT in ccRCC cells by modulating the PI3K/AKT signaling pathway. In turn, the interaction of CXCL13 and CXCR5 and the CCL20-CCR6 axis could promoted ccRCC proliferation and EMT through the PI3K/AKT signaling pathway. The CCL2 from M2 TAMs inhibited autophagy through the MBNL2/Bcl-2/Beclin axis, thereby promoting growth, metastasis and EMT process. Thirdly, exosomes also promote EMT in RCC cells. Exosome-carrying miR-21-5p targeted PTEN-3′UTR, downregulated tumor suppressors, activated AKT signaling, and enhanced tumor cell migration and invasion. Another exosome-carrying miR-342-3p promoted RCC cells progression through the NEDD4L-CEP55 axis

Invasion and metastasis of tumor tissue involve a complicated, multistep procedure, by which tumor cells detach from the primary lesion and colonize various organs via the lymphatic or circulatory pathways. The metastatic mechanisms of tumor cells are complex, involving interactions of tumor cells with other TME cells, dysregulation of cytokine and chemokine expression, modification of tumor cell polarities, and remodeling of the extracellular matrix and the TME. As an essential component in TME immune cells, TAMs play a crucial role in the migration and metastasis of a wide range of malignancies. As previously described, RCC cells act on TAMs through various mechanisms and promote the polarization of pro-tumor M2 TAMs, which further assist the rapid proliferation and angiogenesis of RCC tissue. Similarly, in the RCC TME, chemokines and exosomes produced by M2 TAMs play crucial roles in regulating the advancement and metastasis of RCC cells (Fig. 3).

EMT plays a pivotal role in tumor metastasis. Undergoing morphological changes, epithelial cells obtain the migratory properties of mesenchymal cells, resulting in cell adhesion loss, which is an important function in tumor progression. TAMs can secrete a variety of cytokines, including IL-6, TGF-β and IL-8; induce EMT in tumor cells; and promote tumor metastasis. One study found that TAMs may promote the migration, invasion, and EMT of RCC cells through activation of the IL-6/STAT3 signaling pathway [49], which further indicates the potential of targeting IL-6 in TAMs for the treatment of progressive RCC.

Similarly, it was discovered that various chemokine ligands perform the same function in promoting EMT in the RCC TME. Studies have shown that TAM-derived chemokine (C-C motif) ligand 5 (CCL5) is associated with progression of the ccRCC TME [50]. Significantly upregulated expression of CCL5 in ccRCC has been correlated to the EMT and tumor development. Furthermore, CCL5 knockdown can significantly inhibit cell viability and migration in vitro, as well as prevent the EMT of clear cell renal cell carcinoma (ccRCC) cells by regulating the PI3K/AKT pathway [50]. In addition, APOC1, which promotes M2 TAM polarization, as described above, is also associated with CCL5. M2 TAMs with APOC1 overexpression promotes RCC cell metastasis by producing CCL5 [43]. Another chemokine ligand and its receptor from the CXC chemokine family also promote ccRCC progression. CXCL13 released by M2 TAMs interacts with its receptor CXCR5 to promote ccRCC proliferation, migration, invasion, and EMT [32]. Studies have shown that CXCL13 is a crucial factor in the promotion of EMT in M2 TAMs. Furthermore, the CXCL13/CXCR5 axis increases the expression of vimentin and N-cadherin, promotes EMT, and significantly enhances ccRCC EMT and proliferation in vivo through the AKT signaling pathway [32].

Similarly, M2 TAMs-derived CCL20 is considered to be a contributing chemokine that promotes RCC cell progression [51]. In the TME of RCC, TAM-secreted CCL20 activates cancer cells through AKT activation, which in turn generates EMT and the capacity for migration. It was demonstrated that the CCL20/CCR6 axis, which acts upstream of the EMT marker, is associated with advanced status and significantly shortens overall survival (OS) in RCC patients. CCL2, another chemokine arising from M2 TAMs, promotes tumor growth, metastasis, and EMT processes by inhibiting autophagy [52]. M2 TAMs also induce and promote the malignant properties of EMT in RCC cells by increasing the expression of muscleblind-like protein 2 (MBNL2). MBNL2 restrains beclin-1 dependent autophagy and promotes the invasive capacity of RCC cells. Upregulation of MBNL2 improves the stability of B-cell lymphoma 2 (Bcl-2), thereby contributing to the formation of the beclin-1/Bcl-2 complex, subsequently inhibiting autophagy [52]. This study, in particular, provides a new direction for the development of therapies for RCC.

The above findings suggest that TAMs in the TME can promote RCC cell invasion and migration through the induction of EMT and other pathways. Therefore, targeting M2 TAMs and related chemokines and exosomes may suppress tumor metastasis by modifying tumor cell polarity.

Similar to the chemokines mentioned above, exosomes also promote EMT in RCC cells. RCC tumor-associated M2 macrophage-derived exosomal miR-21-5p targets phosphate and tensin homolog (PTEN-3′ UTR), downregulates the expression of tumor suppressor genes, and activates AKT signaling, thereby enhancing the migration and invasion capacity of tumor cells [53]. Another macrophage-derived exosome carrying miR-342-3p also facilitated RCC cell progression via the NEDD4L/CEP55 axis [54]. High expression of miR-342-3p was detected in M2 TAM extracellular vesicles and specifically inhibited E3 ubiquitin ligase NEDD4L. NEDD4L has been associated with the ubiquitination and degradation of CEP55, which plays a pro-carcinogenic role in RCC. Thus, M2 TAMs-derived miR-342-3p exerts a pro-oncogenic effect by suppressing NEDD4L and subsequently preventing CEP55 degradation and ubiquitination. In addition, M2 TAMs-loaded miR-342-3p could intensely drive the proliferation, migration, and invasion capacity of RCC cells by activating the PI3K/AKT/mammalian target of rapamycin (mTOR) signaling pathway [54].

In addition to the above ligands and exosomes, there are other factors with carcinogenic potential and pro-tumor properties in M2 TAMs. Cathepsin Z (CTSZ) is a member of the cathepsin family, which has been discovered to modulate the adhesion and migratory properties of immune and tumor cells. Macrophage-specific CTSZ has been reported to be associated with EMT activation in the TME [55]. Macrophage-capping protein (CapG) is a recently discovered oncogene associated with multiple cancers. Studies have shown that silencing of CapG can induce cycle arrest and apoptosis of RCC cells, thereby impairing RCC cell proliferation [56]. Moreover, CapG knockdown affects crucial pathways involved in cancer development, including Ras-related C3 botulinum toxin substrate (RAC) protein, cell division cycle 42 (CDC42) gene, and ERK/MAPK signaling pathways [56]. These results suggest that CapG may be a valid biomarker and possible treatment target for RCC. The complement C1q produced by TAMs is also involved in promoting tumor growth [57]. C1q is associated with tissue inflammation and cancer progression, and TAMs are the cell type that produces the most C1q in the TME of ccRCC. Furthermore, this study demonstrated the pro-carcinogenic effects of C1q, with mouse models lacking C1q, C4, or C3 showing reduced tumor growth [57]. C1q also inhibited the activation, proliferation, and cytotoxic properties of CD8 + T cells. Subsequently, the combined effects of complement activation products, inflammation, and T cell exhaustion promote tumor progression [57].

In summary, TAMs play important roles in RCC progression and are a robust promoter of immunosuppression properties within the TME.

#### Therapy resistance and immune suppression of RCC affected by TAMs

Increasing evidence and research (from preclinical models and trials) are demonstrating the involvement of PD-1/PD-L1 in maintaining the immunosuppressive environment of various cancers [58]. Studies have identified the effectiveness of targeted immune checkpoint inhibitors (ICIs) in reversing immunosuppression and inhibiting tumor growth [59]. The activation of PD-1/PD-L1 as one of the mechanisms of tumor immune escape has been extensively studied, and studies have elucidated the involvement of TAMs in the activation process [59, 60].

A regression analysis employed by one study revealed that TAMs and T regulatory cells (Tregs) were negatively correlated with survival in renal cancer [61]. Infiltration of M2 TAMs and upregulation of IL-10 expression disrupt T-cell function, causing an imbalance in tumor immunity. Research has shown that in the TME, TAMs promote FOXP3 and cytotoxic T-lymphocyte associated protein 4 (CTLA-4) expression in T cells, which happens to be an immune escape mechanism of kidney cancer [62]. The induced expression of FOXP3 in Tregs promotes the immune escape of RCC, and the upregulation of CTLA-4 expression in autologous T cells promotes cancer immunosuppression, and the co-occurrence of these factors has been associated with a worse prognosis in renal cancer patients [62]. Meanwhile, this study demonstrated that TAMs promote immunosuppression through metabolism-related mechanisms. TAMs enhance arachidonic acid production by upregulating 15-lipoxygenase 2 (15-LOX-2) expression, which promotes IL-10 and CCL2 production. This pathway affects the immune function of macrophages and other immune cells, leading to local immunosuppression and tumor escape. Another study [63] also showed the relevance of TAMs to FOXP3. CCR8-positive TAMs induced FOXP3 expression in autologous T cells via the signal transducers and activators of transcription (STAT) pathway, which had an impact on tumor immunosuppression. Furthermore, this study indicated that the CCR8/CCL1 axis may be associated with cancer-related inflammation and immune escape.

Tumor stem cells have the capacity for self-renewal, which is associated with tumor progression, metastasis, and recurrence, and are also considered to be an important cell population for tumor therapeutic resistance [64]. CD44 can be utilized as a marker for tumor stem cells, and it has been demonstrated that increased infiltration of TAMs is correlated with upregulation of CD44 expression and the generation of tumor stem cells. It was shown in a co-culture model of TAMs with human RCC cell lines that TAMs mediate NF-kB activation through TNF-α secretion, leading to CD44 overexpression in RCC cells [64]. Another study performed experiments in in vivo and in vitro models and found that TAMs increase the number of tumor stem cell populations by altering AKT/mTOR signaling, which leads to treatment resistance [65]. This alteration also promotes EMT in RCC, which increases the invasive capacity of RCC cells, leading to tumor progression and metastasis. Another study [66] found that infiltrating TAMs induced RCC cells to develop resistance to sunitinib and mTOR inhibitors through high expression of T-cell immunoglobulin and mucin domain-containing protein 3 (TIM-3) expression, and that TIM-3 on tumor and myeloid cells synergistically promoted the tumorigenic activity and enhanced the stem cell properties of RCC cells.

## Clinical application of TAMs in RCC immunotherapy

### Application of TAMs in the diagnosis and prognosis of RCC

As research on the association between TAMs and malignancies progresses, laboratory findings of TAMs have been introduced in enter clinical applications, indicating that TAMs have become potential diagnostic, prognostic, and therapeutic biomarkers for various cancers. Research suggests that higher infiltration levels of TAMs are correlated with worse clinical outcomes of RCC patients and may serve as a biomarker of prognosis and treatment response.

A study suggested that M1 TAMs infiltrating the TME have an impact on cancer prognosis and clinical outcome prediction. M1 TAMs were positively correlated with TMN staging and histological grading of cancer, and were an independent predictor for OS and disease-free survival (DFS) in patients with RCC [67]. Another study found that CTSZ was probably a macro-specific marker in RCC, with macro-specific CTSZ correlating with EMT initiation, cell cycle characteristics, and greater levels of TAM and B cell infiltration in the TME [55]. These results indicated that high expression levels of CTSZ in ccRCC patients treated with anti-PD-1 immunotherapy could be considered a biomarker of prognosis and therapeutic efficacy.

Three other studies combined novel molecules with CD163 + TAMs to improve the prognostic prediction accuracy for RCC patients. One study found that the expression level of ring finger protein 43 (RNF43) was negatively correlated with the extent of CD163 + TAM infiltration in ccRCC, and was strongly correlated with the TNM stage and clinical outcome of RCC patients [68]. Furthermore, they found that the combination of intratumoral RNF43 expression, CD163 + TAM infiltration, and TNM staging could predict postoperative prognosis through time-dependent C-index analysis and a nomogram [68]. Similarly, another study indicated that the combination of intratumoral ubiquitin-protein ligase N-recognin 5 (UBR5) and CD163 had a higher C-index value than the use of UBR5, CD163, or TNM stag alone in predicting prognosis [69]. Another study observed that Ras-association domain family member 10 (RASSF10) expression was negatively correlated with RCC patient prognosis and suggested that combining RASSF10 and CD68 (or CD163) showed better efficacy in predicting the OS and DFS for postoperative patients.

In addition, Shen et al. [31] conducted a meta-analysis on the prognostic value of TAMs in RCC patients, finding that high TAM expression indicates poor prognosis and occurs more frequently in RCC patients with clinicopathological characteristics of advanced stage, such as higher nuclear grading, tumor necrosis, and advanced Union for International Cancer Control TNM classification. Also, they found that M2 TAMs serve as a risk factor for worse prognosis in ccRCC, but are also a potential therapeutic target. Using selective small molecule inhibitors to block the M2 TAMs–related pathway may be a promising method for reducing the infiltration of M2 TAMs for RCC patients [31]. Sophie et al. [70] emphasized the potential impact of TAM HIF-1α as an independent prognostic agent for RCC. HIF-1α is mainly expressed in TAMs, and TAM-derived HIF-1α is closely correlated with worse tumor stage and development, and independently related to poor OS and outcomes in RCC patients. In addition, HIF-1α is an important predictor of decreased OS in multi-variable settings, with its elevated expression being closely related to anti-angiogenic therapy resistance. These results validated that HIF-1α inhibitors could be a potential method for targeting the pro-cancer properties of TAMs in RCC patients.

Recently, a study investigated the correlation between macrophages migration inhibitory factor (MIF) expression and prognosis of ccRCC patients [71]. They found that the negative expression of MIF may be an independent prognostic factor for ccRCC patients, resulting in poor DFS and disease-specific survival. Liu [72] et al. constructed a prognostic model associated with macrophage differentiation through Cox analysis. The model consisted of six macrophage differentiation-elated genes (MDGs), including CD14, ABCG1, KDF1, TGFA, HAVCR2, and KITLG. The mRNA expression levels of MDGs in clinical ccRCC tissues were shown to exhibit higher expressions of CD14, ABCG1, TGFA, and HAVCR2 but lower expressions of KITLG and KDF1 in the tumor samples than in the adjacent control samples. This prognostic model has high accuracy in predicting the survival rate of ccRCC patients, suggesting that this prognostic model might be useful for studying biomarkers and assessing the prognosis of advanced RCC [72].

### Application of TAMs in the targeted therapy of RCC

As research on tumor immunotherapy progresses, the role of immune cells in the TME is increasingly becoming a central research focus. An increasing number of studies have emphasized the important role of M2 TAMs in RCC development and progression. Thus, methods to remove M2 macrophages or convert them into carcinogenic M1 TAMs will be a valuable direction for future RCC immunotherapy. Although macrophages-targeted therapies for RCC are still in their infancy, TAM-targeted therapies for other common tumors have been progressing gradually, and these studies may pave the way for RCC-specific immunotherapies. Studies found that in colorectal cancer, colony-stimulating factor-1 receptor (CSF1R) inhibitors specifically inhibited the survival of protumor M2 TAMs while having little effect on the growth of anti-tumor M1 TAMs, resulting in an increased M1/M2 ratio [73, 74]. This reversed the immune-suppressive effects of the TME, resulting in suppression of colon cancer development [74]. In certain other solid tumors, such as prostate cancer and osteosarcoma, macrophage-targeting strategies on CSF1R have also shown efficacy in suppressing tumor development [75, 76]. Moreover, targeting the CD47-SIRPα axis was significantly effective in inhibiting tumor progression in an ovarian cancer model, which was involved in macrophage-mediated phagocytosis in vitro. [77]. The above advancements in research will facilitate the improvement of macrophage-targeted therapies for RCC in the future.

**Fig. 4:**
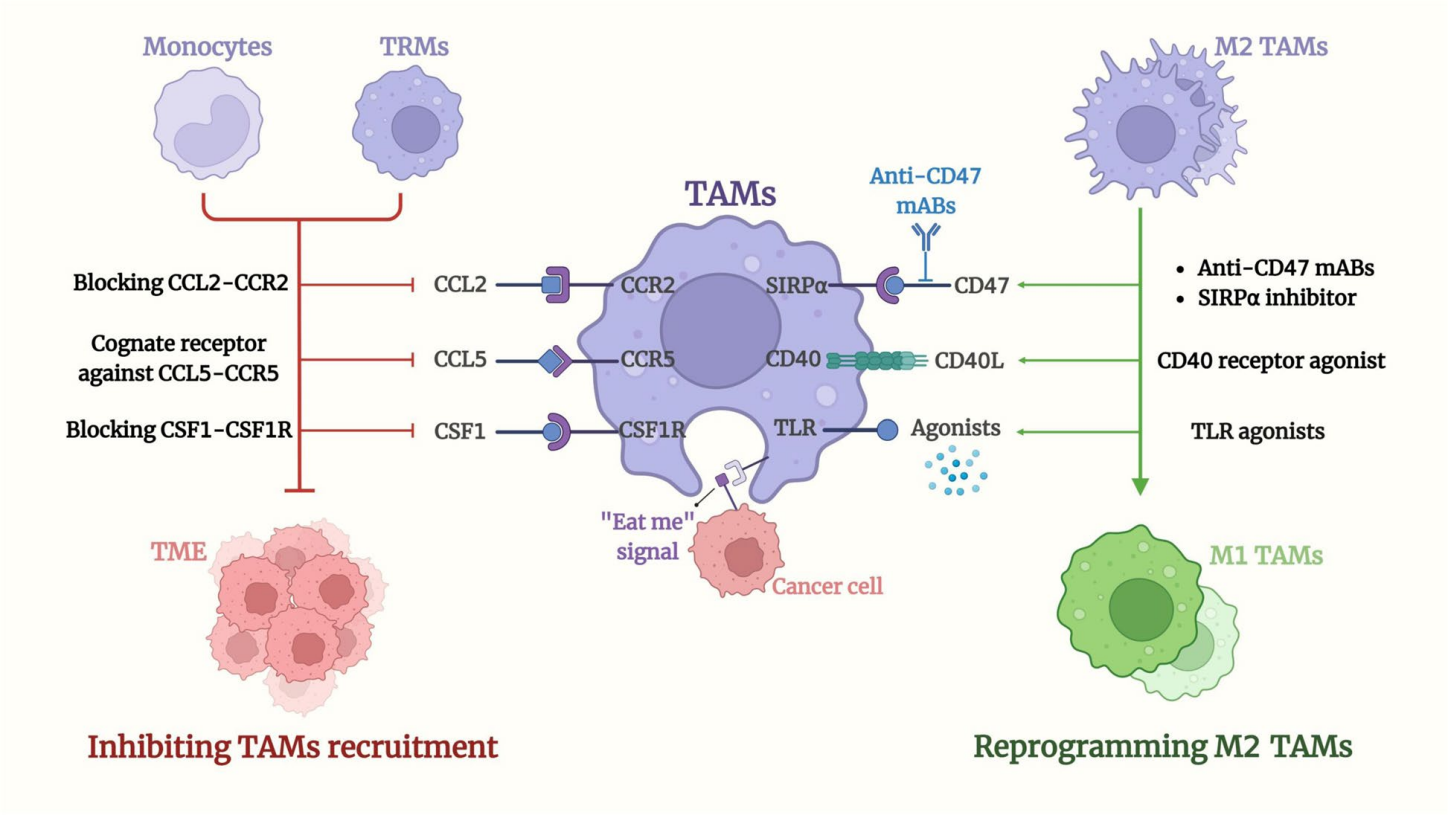
Targeted therapy of TAMs in RCC. The therapeutic approaches to modulate TAMs function can be divided into two categories: inhibiting recruitment of TAMs in the TME; And polarizing from M2 TAMs to M1 TAMs. By blocking the signaling pathways associated with recruitment and polarization of TAMs, recruitment to the TME by monocytes and TRMs can be inhibited. Inhibition of CCL2-CCR2 and CSF1-CSF1R can suppress the interaction between tumor cells and TAMs, thereby reducing the infiltration of M2-like immunosuppressive macrophages into the TME. The application of CCR5 homologous receptors inhibited the CCL5-CCR5 axis, thus suppressing recruitment of M2 TAMs in the TME. Reprogramming M2 TAMs into M1 TAMs reversed the pro-tumor phenotype of TAMs and re-endowed them with positive defense activity and anti-tumor functions. One approach to re-education is to target the CD47-SIRPα pathway, in which CD47 mABs modulate the transformation to M1 TAMs and SIRPα inhibitors also stimulate the activation of anti-tumor TAMs. Another approach to transform M2 TAMs is the use of a CD40 receptor agonist, which not only transforms TAMs but also promotes T cell activation. Similarly, TLR agonists effectively transform TAMs to M1 type, leading to an inflammatory cytokine response and effective inhibition of solid tumors

Various strategies for removing TAMs from the TME have been explored, and due to the dual activities of TAMs, studies on the re-education or de-differentiation of pro-tumorigenic TAMs have gradually increased. By utilizing the high degree of macrophage plasticity, scientists have transformed immune-suppressive M2 TAMs into immune-stimulating M1 TAMs, thereby inducing anti-tumor effects. [78, 79]. This therapy method can also block the effects produced by M2 TAMs, such as preventing angiogenesis, cancer progression, and immune escape. It also works synergistically with other treatments, thereby enhancing the efficacy of other cancer therapies, such as radiotherapy, chemotherapy, and ICIs. Therapies that modulate the polarization and function of TAMs could be divided into two categories: The first eliminates TAMs or inhibits TAM recruitment from TME; the second repolarizes TAMs from M2 TAMs into M1 TAMs (Fig. 4 and Tables 1 and 2).

**Table 1:** Cytokines and pathways associated with therapeutic approaches to TAMs in biological development

**Therapeutic approaches**	**Cytokines/Pathways**	**Mechanisms/Effects**
Inhibiting TAMs recruitment	Blocking CXCL12, CX3CL1	Suppressing TAMs recruitment into the TME.
	Inhibiting CCL2	Inhibiting bone marrow monocyte infiltration and TAMs polarization.
	Inhibiting CCR2	Inhibiting macrophage polarization and cancer metastasis.
	Targeting CSF-1R pathway	Inhibiting TAMs recruitment and increasing proportion of CD8+/CD4+ T cells.
Reprogramming M2 TAMs	Anti-CD47 mAb	Re-educating TAMs with anti-tumor properties by manipulating the CD47–SIRPα axis.
	SIRPα-blocking antibodies	Combination with CSF-1R inhibitors stimulated the anti-tumor macrophages activation.
	CD40 agonist	Reprogramming TAMs and converting them into tumor-resistant M1 TAMs.
	TLR3 stimulators	Inhibiting tumor development by producing high levels of TNF-α and NO.

**Table 2:** Compounds and pathways associated with therapeutic approaches to TAMs in clinical development

**Therapeutic approaches**	**Compounds**	**Cytokines/Pathways**	**Mechanisms/Effects**
Inhibiting TAMs recruitment	Carlumab, CNTO 888	Inhibiting CCL2-CCR2 pathway	Inhibiting macrophage polarization in the TME.
	BMS-813160	Dual CCR2/CCR5 antagonist	Inhibiting both TAMs function and MDSCs activity.
	Maraviroc	CCR5 homologous receptor	Reducing M2 TAMs recruitment in combination with conventional chemotherapy.
	Emactuzuma b (RG7155)	Anti-CSF-1R antibody	Specifically inhibiting CSF-1R dimerization and removing M2 TAMs.
	Pexidartinib (PLX3397)	CSF-1/CSF-1R signaling inhibitor	Suppressing M2 TAMs polarization in vitro and depleting TAMs in the TME.
Reprogramming M2 TAMs	Imiquimod (R-387)	TLR7 agonist	Re-educating the M2-like polarization of TAMs.
	Lefitolimod	TLR9 agonist	Improving M1 TAMs functions and inducing other responses against tumors.

#### Inhibition of the recruitment of TAMs

As previously described, monocytes derived from the bone marrow and blood circulation are recruited by the TME, then differentiate into two major classes of TAMs. This phenomenon is mediated by a range of cytokines, chemokines, and signaling pathways [22], such as CSF-1, VEGF, and complement components. Under the chemotactic influence of these mediators, recipient macrophages are recruited to the tumor site. Inhibiting the aggregation and polarization of M2 TAMs by blocking these cytokines and pathways has become one of the main approaches for anti-TAM recruitment therapy.

As one of the key factors in TAM recruitment, chemokines have received extensive attention. Studies have shown that blocking certain chemokines, like CXCL12, CX3CL1, and their corresponding receptors, could suppress TAM recruitment into the TME [80, 81]. In turn, the interactions between chemokines and their receptors have been intensively investigated. The interplay between CCL2 and CCR2 has an important role in the recruitment and polarization of TAMs. These two chemokines also exert immunosuppressive effects by participating in the induction and activation of other immune components, including myeloid-derived suppressor cells (MDSCs) and Tregs [82]. Studies have shown that inhibition of CCL2 reduces bone marrow monocyte infiltration and TAM polarization, thereby slowing down tumor growth in various cancers, such as lung cancer, bladder cancer, and hepatocellular carcinoma (HCC) [83]. In addition, using anti-CCL2 antibodies attenuated radiation resistance in pancreatic cancer models [84, 85]. In the case of CCR2, inhibition of CCR2 was also effective in reducing cancer metastasis in preclinical models [86]. Inhibition of the CCL2-CCR2 pathway is, therefore, an important method for inhibiting macrophage polarization in the TME, and relevant clinical studies have been conducted (carlumab [CNTO888]) [83, 86]. In addition to targeting CCR2 alone, the dual blockade of CCR2 and CCR5 (BMS-813160) has also been investigated, as this may inhibit both TAM function and MDSC activity [83].

Furthermore, CCL5, the ligand of CCR5, has shown a tumor-promoting effect in the TME. CCR5, which can be generated by tumor cells and TAMs, recruits macrophages and skews them towards functions that are favorable to the tumor phenotype [87]. Through applying the CCR5 cognate receptor, recruitment of M2 TAMs can be inhibited, thereby inhibiting the TME. In a phase 1 clinical trial, maraviroc (UK427857), a homologous receptor for CCR5, in combination with conventional chemotherapy, showed promising therapeutic efficacy by reducing the recruitment of M2 TAMs [88].

Similar to CCR5, many additional tumor-secreted factors, including VEGF and CSF-1, are also essential for macrophage recruitment. As the receptor of CSF-1, CSF-1R is highly expressed in TAMs. By binding to CSF-1R, CSF-1 produced by tumor cells promotes TAM recruitment and polarization. Therefore, blocking the CSF-1/CSF-1R axis could be effective for inhibiting the association between TAMs and tumor cells. Several CSF-1R monoclonal antibodies (mAbs) have shown promising anti-tumoral effects in preclinical studies [89, 90]. Emactuzumab (RG7155) is a mAb to CSF-1R that enhances T cell-mediated anti-tumor immune responses via a mechanism of specific inhibition of CSF-1R dimerization and consequent clearance of M2 macrophages expressing the CD163 antigen and CSF-1R [90].

CSF1R inhibitors play a similar role, and pexidartinib (PLX3397) has been shown to reduce tumor cell proliferation and produce tumor ablation in a variety of cancer models, including gliomas and breast and lung cancers [91, 92]. In addition to inhibiting the recruitment of macrophages, targeting the CSF-1R signaling pathway could also lead to an increased proportion of CD8+/CD4 + T cells [93]. Various CSF1R blockers, such as LY3022855 mAb (IMC-CS4), DCC3014, Plexxikon, and AMG820 mAb (lacnotuzumab [MCS110]), are undergoing diverse preclinical and clinical evaluations, but with conflicting results to date [75, 94, 95].

In conclusion, these approaches targeting TAM recruitment and polarization reduce immunosuppressive M2 TAM infiltration of the TME, thereby enhancing the effect of immunotherapy in concert with other conventional treatments.

#### Reprogramming of M2 TAMs

As previously described, M2 TAMs exhibit a pro-tumor subtype in the TME and induce tumor progression and immune escape. Therefore, reprogramming M2 TAMs to convert into immune-induced and anti-tumor M1 TAMs is also a potential therapeutic method.

In various cancer models, targeted therapy of reprogramming TAMs intends to re-educate M2 TAMs with positive defense activity and anti-tumor functions, such as direct tumor cell killing, angiogenesis and metastasis suppression, and enhancement of adaptive immune responses [96]. To reverse the anti-tumor properties, it is necessary to regain the phagocytic capacity of macrophages. In addition, reprogramming TAMs in the TME is also promising for enhancing the efficacy of other anti-tumor therapies that are in current clinical use, such as ICIs or CAR T cells [97]. Given that targeting TAMs is considered essential for cancer therapy, researchers have investigated several mAbs and toll-like receptors (TLRs) to manipulate TAM polarization or reprogramming in the TME.

Among the mAbs studied, the anti-CD47 mAb and the CD40 agonist have demonstrated agonistic properties that reprogram TAMs to produce potent anti-tumor activities. An important approach in re-educating TAMs with anti-tumor properties using mAbs is to manipulate the CD47-SIRPα axis. As an important tumor antigen that affects the onset and progression of various cancers, CD47 interacts with SIRPα to release a certain signal to escape phagocytosis [98].

In myeloid cells, SIRPα, belonging to the immunoglobulin superfamily, is highly expressed and plays an important role in the maintenance of tissue homeostasis [98]. The mAb of CD47 targets the CD47-SIRPα pathway, and converts TAMs into anti-tumor properties. This measure also induces antibody-dependent cellular phagocytosis mediated by SIRPα, thereby improving M2 function [99]. For instance, the use of CD47 blockers can enhance the phagocytosis of M2 TAMs and inhibit tumor advancement in endometrial cancer [100].

In addition to targeting CD47, it has been found that SIRPα–blocking antibodies combined with CSF-1R inhibitors can stimulate the activation of anti-tumor macrophages [101]. This combination therapy not only blocks the CD47-SIRPα pathway, but also hinders the recruitment of neo-TAMs by inhibiting CSF-1R [102]. Another approach to transforming M2 TAMs is to activate CD40 expressed on the surface of macrophages, which can regulate TAM polarization. The CD40 agonist has been shown to be effective in inhibiting prostate cancer progression and can make previously drug-resistant tumors sensitive to chemotherapy [103]. Activation of the CD40 receptor can reprogram TAMs and transform them into tumor-resistant M1 macrophages [104]. CD40 ligands are predominantly expressed on T cells, and research has indicated that the interaction between CD40 on macrophages with its ligands can promote T cell activation [105]. In addition, similar to CD-47 combination therapy, applying CD40 receptor agonists after inhibiting the CSF-1 receptor can remarkably enhance T-cell activity, thereby boosting anti-tumor immune responses [106].

Another factor influencing TAM reprogramming is TLRs, which play a crucial role in coordinating the immune system as an innate immune pattern recognition receptor. [107]. TLRs can be activated by viral nucleic acids and LPS, thereby effectively transforming TAMs into M1 macrophages, which leads to an inflammatory response and potent inhibition in the TME [108–110]. TLR3, TLR4, TLR7/8, and TLR9 agonists are the most commonly used mediators targeting TLRs [111]. Among them, the TLR7 ligand imiquimod (R-837) is a clinically approved TLR agonist that exerts anti-neoplastic activity in some cancers, including melanoma, basal cell cancer, and breast cancer [112, 113]. In breast cancer patients with skin metastasis, imiquimod (NCT00899574) showed partial immunotherapeutic responses [114]. Nanoparticle-loaded TLR3 stimulators have been shown to have inhibitory effects on tumor development by producing high levels of TNF-α and nitric oxide (NO) [115]. TLR9 agonist lefitolimod (MGN1703) not only improved M1 TAMs, but also induced other responses against tumors [116, 117], and has been the topic of several clinical studies [118]. A study based on immunotherapy resistance reported that nanogels coated with TLR agonists and long peptide antigens could target TAMs and transform pro-tumor M2-TAMs into anti-tumor M1-TAMs [119].

### Metabolic regulation of TAMs

TAMs are of interest due to their high degree of functional plasticity, which is influenced by a variety of biomolecules, whereas metabolites determine the polarization of their functional phenotype [100]. During the anti-inflammatory process of M1 TAMs, lactate accumulation occurs due to increased glycolysis and the Warburg effect. It has been shown that lactate and lactylation exert an inducing effect on the polarization process of TAMs.

Increased histone lysine lactylation induces the expression of the homeostatic gene Arg1, which promotes the activation of M2-associated genes in M1 TAMs and may contribute to the repair of tissue damage [120]. In addition, lactate may regulate M2 macrophage polarization by specifically recognizing G protein coupled receptor 132 (GPR132). Additionally, lactate specifically recognizes GPR132, the activation of which contributes to inflammation and tumor growth. Furthermore, it has been shown that peroxisome proliferator-activated receptor γ (PPARγ) inhibits the expression of GPR132 proteins in macrophages, so targeting the lactate-PPARγ/GPR132 receptor-related pathway may have an inhibitory effect on the pro-tumor effects of TAMs [121]. Lipid accumulation in TAMs can also lead to differentiation into pro-tumorigenic phenotypes. The activation of caspase-1 was found to promote the accumulation of lipids. As a result, caspase-1 inhibitors, such as VAD, YVAD, and NCX-4016, are able to reprogram TAMs into an anti-tumor phenotype and prevent tumor growth in vivo.

Activation of the mTOR pathway is common in cancer and could directly impact the modulation of TAM metabolism in a number of pathways [122]. mTOR is a serine/threonine kinase with two main complex forms: mTORC1 and mTORC2 [123]. The application of the mTORC1-selective blocker rapamycin not only directly inhibited tumor cell activity, but also polarized TAMs into an anti-tumor M1 TAMs [124].

TAM-produced cyclooxygenase-2 (COX-2) promotes the conversion of M1 TAMs into M2 TAMs and produces immunosuppressive prostaglandin E2 (PGE2) [125]. Therefore, COX-2 and PGE2 became targets for TAM inhibition [125]. For example, in an HCC model, the expression of M2 TAMs markers was reduced, and tumor growth was significantly inhibited by the application of COX-2 inhibitors compared to controls [126].

Although there is substantial evidence that targeting TAM metabolism can effectively inhibit tumor growth in tumor models, further preclinical research is still needed to discover the potential for reprogramming TAM epigenetic and metabolic networks as a means of enhancing the efficacy of immunotherapy.

## Conclusion

This review investigated the interaction of TAMs with RCC, highlighting the M1/M2 TAM polarization process, their impact on tumorigenesis and development, and their potential application for targeted immunotherapy. TAMs exhibit significant heterogeneity and play important roles in RCC. First, we presented the relationship between macrophages and TAMs, and discussed the various stimuli and signals related to TAM polarization. Second, we highlighted the different roles of TAMs in tumorigenesis and cancer advancement, then briefly described the relationship between TAMs and tumor immunosuppression and drug resistance. Polarized M2 TAMs dominated the promotion of tumor proliferation, angiogenesis, invasion, drug resistance, and immunosuppressive microenvironment. Last, on the basis of these studies, we concluded that blocking TAM recruitment in the TME, or repolarizing M2 TAMs may be helpful in the treatment of patients with progressive RCC.

## Data Availability

Not applicable.

## Abbreviations

RCC: Renal cell carcinoma
TME: Tumor microenvironment
TAMs: Tumor-associated macrophages
LPS: Lipopolysaccharide
IFN-γ: Interferon-γ
TNF-α: Tumor necrosis factor-α
GM-CSF: Granulocyte macrophage-colony stimulating factor
IL: Interleukin
PD-L1: Programmed death-ligand 1
TGF-β: Transforming growth factor-β
VEGF: Vascular endothelial growth factor
ROS: Reactive oxygen species
TCA: Tricarboxylic acid
HIF-1α: Hypoxia-inducible factor-1α
FAO: Fatty acid oxidation
JMJD3: Jumonji structural domain-containing protein 3
IME: Immune microenvironment
TNM: TNM Classification of Malignant Tumors
CXCL13: C-X-C motif chemokine ligand 13
EMT: Epithelial mesenchymal transition
LncRNAs: Long non-coding RNAs
STAT3: Signal transducer and activator of transcription 3
CircRNAs: Circular RNAs
FOX: Forkhead box
TRAF2: Tumor necrosis factor receptor-associated factor-2
RBM15: RNA-binding motif protein 15
BLTR2: Leukotriene B4 receptor 2
CNTNAP1: Contactin-associated protein 1
AQP9: Aquaporin 9
APOC1: Apolipoprotein C1
AIM2: Absent in melanoma 2
VM: Vasculogenic mimicry
3′UTR: 3′-untranslated region
TIMP2: Tissue inhibitor of metalloproteinases 2
HuR: Human antigen R
IGF-1: Insulin-like growth factor 1
CCL: Chemokine (C-C motif) ligand
ccRCC: Clear cell renal cell carcinoma
OS: Overall survival
MBNL2: Muscleblind-like protein 2
Bcl-2: B-cell lymphoma 2
PTEN: Phosphate and tensin homolog
mTOR: Mammalian target of rapamycin
CTSZ: Cathepsin Z
CapG: Macrophage-capping protein
RAC: Ras-related C3 botulinum toxin substrate
CDC42: Cell division cycle 42
ICIs: Immune checkpoint inhibitors
Tregs: T regulatory cells
CTLA-4: Cytotoxic T-lymphocyte associated protein 4
15-LOX-2: 15-lipoxygenase 2
STAT: Signal transducers and activators of transcription
TIM-3: T-cell immunoglobulin and mucin domain-containing protein 3
DFS: Disease free survival
RNF43: Ring finger protein 43
UBR5: Ubiquitin-protein ligase N-recognin 5
RASSF10: Ras association domain family member 10
MIF: Migration inhibitory factor
MDGs: Macrophage differentiation-elated genes
CSF1R: Colony-stimulating factor-1 receptor
SIRPα: Signal regulatory protein α
MDSCs: Myeloid-derived suppressor cells
HCC: Hepatocellular carcinoma
mAbs: Monoclonal antibodies
TLRs: Toll-like receptors
GPR132: G protein coupled receptor 132
PPARγ: Peroxisome proliferator-activated receptor γ
COX-2: Cyclooxygenase-2
PGE2: Prostaglandin E2
